# Complete genome sequence of a marine roseophage provides evidence into the evolution of gene transfer agents in alphaproteobacteria

**DOI:** 10.1186/1743-422X-8-124

**Published:** 2011-03-17

**Authors:** Sijun Huang, Yongyu Zhang, Feng Chen, Nianzhi Jiao

**Affiliations:** 1 State Key Laboratory of Marine Environmental Science, Xiamen University, Xiamen 361005, PR China; 2 Institute of Marine and Environmental Technology, University of Maryland Center for Environmental Science, Baltimore, MD 21202, USA; 3 Key Laboratory of Urban Environment and Health, Institute of Urban Environment, Chinese Academy of Sciences, Xiamen 361012, PR China

## Abstract

Roseophage RDJLΦ1 is a siphovirus isolated from South China Sea on *Roseobacter denitrificans *OCh114. Its virion encapsulates 62.7 kb genome that encodes 87 gene products. RDJLΦ1 shares similar genome organization and gene content with the marine bacteriophage ΦJL001 and *Pseudomonas *phages YuA and M6, which are different from those of typical λ- or Mu-like phages. Four hallmark genes (ORFs 81 to 84) of RDJLΦ1 were highly homologous to RcGTA-like genes 12 to 15. The largest gene (ORF 84) was predicted to encode a tail fibre protein that could be involved in host recognition. Extended phylogenetic and comparative genomic analyses based on 77 RcGTA-like element-containing bacterial genomes revealed that RcGTA-like genes 12 to 15 together appear to be a conserved modular element that could also be found in some phage or prophage genomes. Our study suggests that RcGTA-like genes-containing phages and prophages and complete RcGTAs possibly descended from a same prophage ancestor that had diverged and then evolved vertically. The complete genome of RDJLΦ1 provides evidence into the hypothesis that extant RcGTA may be a prophage remnant.

## Findings

Viruses are the most abundant entities of the world's oceans, ranging from ~3×10^6 ^to ~10^8 ^viruses per ml [1]. Bacteriophages (viruses that infect bacteria) are known to play an important role in regulating the species composition of bacteria [1-4] and in the host evolution through phage-mediated horizontal gene transfer [5-7]. Bacteria in the *Roseobacter *clade (roseobacter hereafter) are abundant, and typically comprise 10-20% of marine bacterial communities [8,9]. More than 30 genomes of representative roseobacters have been sequenced [10], and the genomics studies also showed that nearly all roseobacter genomes carry a conserved gene transfer agent (GTA) gene cluster of RcGTA (GTA producted by *Rhodobacter capsulatus*) type [10-14] which could assemble a phage-like particle that transfers random pieces of genome DNA from producing cells to recipient cells through a generalized transduction-like process [15]. The widespread occurrence of RcGTA was hypothesized to be a potential efficient mechanism for horizontal gene transfer [13].

Up to date, only a limited number of roseophage (phage that infects roseobacter) genomes have been reported [16-18], which were all in *Podoviridae *family. In this study, we presented the genome sequence of Roseophage RDJLΦ1 that infects *Roseobacter denitrificans *OCh114. RDJLΦ1 was isolated from the South China Sea surface water (17.597°N, 116.029°E) collected in September 2007 as previously described [19]. RDJLΦ1 was characterized as a host-specific siphovirus, which has an isometric head and a long, flexible, non-contractile tail, and belongs to *Siphoviridae *family, *Caudovirales *order [19]. RDJLΦ1 is a lytic phage with burst size of *ca*. 203 and latent period of *ca*. 80 min [19]. This is the first presented genome of a siphovirus infecting marine *Roseobacter*.

**Figure 1:**
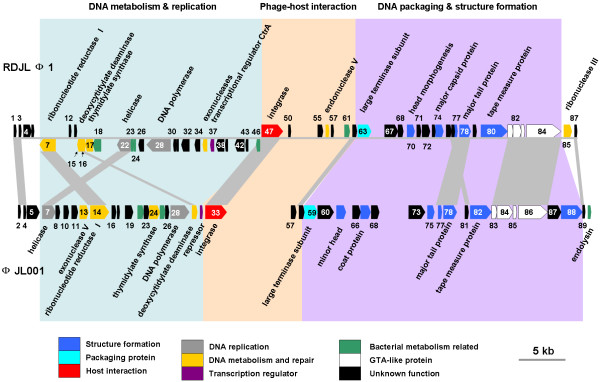
**Genome map of roseophage RDJLΦ1**. Bacteriophage ΦJL001 [NC_006938] was shown for reference. Generally, only ORFs of significant BLAST hit (E ≤ 0.001) were shown. ORFs were depicted by leftward (below the gay line for RDJLΦ1) or rightward (above the grey line for RDJLΦ1) oriented arrows indicating the direction of transcription. Homologues between RDJLΦ1 and ΦJL001 were indicated by grey shadows. Three functional modules were highlighted by blue, pink and purple background. The genome sequence of RDJLΦ1 was deposited in GenBank with accession number [HM151342].

The circularly assembled genome of RDJLΦ1 comprises 62,668 bp, with a G+C content of 57.9%, strongly resembling the G+C average (58.0%) of its host. The whole genome was sequenced by using shotgun library method with 7-fold coverage. In total, 87 open reading frames (ORFs) were predicted from the genome using Glimmer [20] and GeneMark [21] (Additional file 1, Figure 1). No tRNA sequences were identified using the tRNAscan-SE program [22]. Fifty-five gene products have homologous sequences in NCBI non-redundant protein database, whereas only 24 of them have predicted functions. Thirty-eight ORFs are homologous to genes in bacteriophages. Among them, 15 ORFs were homologues of genes in another siphovirus ΦJL001 that infects an uncharacterized marine sponge-associated alphaproteobacterium, JL001 [23] (Figure 1, indicated by grey shadows). Most of these homologues between RDJLΦ1 and ΦJL001 scatter in similar loci of the genomes. Based on the sequence homology, RDJLΦ1 is most closely related to ΦJL001 among all the known phage genomes. Moreover, 7 of those 15 ORFs are also homologous to genes from *Pseudomonas *phage YuA [24] and/or M6 [25]; YuA and M6 are 91% identical to each other at the DNA level [24]. Five phage structural proteins, which are tail fibre protein (gp84), tail tape measure protein (gp80), major tail protein (gp78), major capsid protein (gp74) and an unknown structural protein (gp67), could be assigned based on the previously reported proteome analysis (Table 1; the five proteins and corresponding SDS-PAGE bands were predicted based on comparing their molecular weights and bands' staining intensity) [19].

**Table 1:** Phage RDJLΦ1 structural protein assignment based on the previously reported SDS-PAGE analysis [19]

SDS-PAGE band	MW (kDa)	ORF no.	Predicted product
A	147.6	84	tail fibre
B	107.9	80	tail tape measure protein
C	53	78	major tail protein
D	52.7	67	unknown structural protein
E	37.7	74	major capsid protein

SDS-PAGE: sodium dodecyl sulfate-polyacrylamide gel electrophoresis;

MW: molecular weight.

The RDJLΦ1 genome can be divided into three modules: (i) DNA metabolism and replication, (ii) phage-host interaction, (iii) DNA packaging and structure formation (Figure 1). This type of genome organization is similar to those of ΦJL001, YuA and M6, but differs from those of λ- and Mu-like phages [24]. RDJLΦ1, ΦJL001, YuA and M6 also share the similar gene contents in the three modules. No lysis genes were predicted in RDJLΦ1 genome, whereas lysis cassettes including four genes (endopeptidase *Rz*, embedded *Rz1*, holing and endolysin) were found in YuA and M6 genomes [24]. Phylogenetic analysis based on terminase large subunit (TerL) protein showed that RDJLΦ1 fell into the "P22-like headful" cluster and was closely related to *Salmonella *phages ES18, E1 and *Listonella *phage ΦHSIC (Additional file 2). Despite the factor that RDJLΦ1, YuA, M6 and ΦJL001 share certain genomic similarity, temperate phages YuA, M6 and ΦJL001 clustered closely together but distantly to RDJLΦ1. Phages that infect marine, even aquatic, bacteria are isolated and characterized less frequently than terrestrial phages, resulting in the difficulty in their taxonomic classifications.

**Figure 2:**
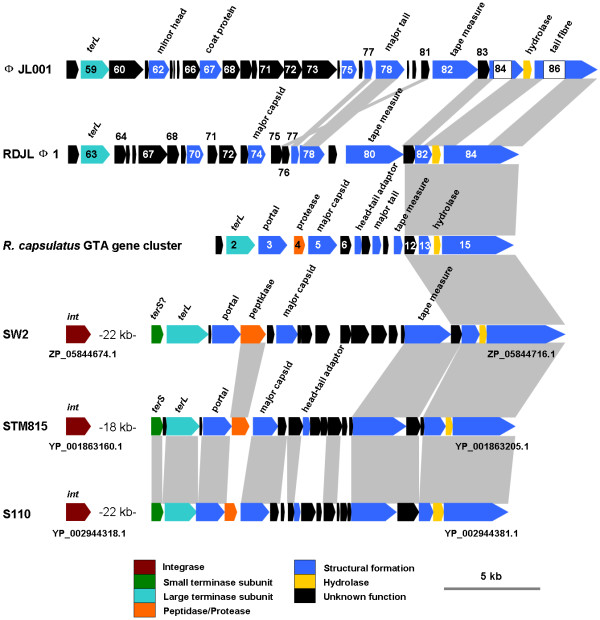
**RcGTA-like gene structure of roseophage RDJLΦ1**. For comparison, partial genome of bacteriophage ΦJL001, prophage-like gene structures in *Rhodobacter *sp. SW2 [NZ_ACYY00000000], *B. phymatum *STM815 plasmid pBPHY01 [NC_010625] and *V. paradoxus *S110 [NC_012791] and GTA gene cluster of *R. capsulatus *[AF181080] were shown. Homologues were indicated by grey shadows.

Four ORFs (81, 82, 83 and 84) of RDJLΦ1 are homologous to RcGTA genes 12, 13, 14 and 15, respectively [12,15] (Additional file 1, Figure 2). gp81 is most closely related to the glycoside hydrolase which could also be the putative product of RcGTA gene 12. ORF 82 was identified as a homologue of RcGTA gene 13 which encodes a structural protein detected by proteomic approaches [26]. ORF 83 contains a phage-related cell wall peptidase domain and was predicted to encode a hydrolase belonging to the NlpC/P60 superfamily. The product of its homologue in RcGTA (gene 14) was not found in proteome [26]. Previously, it was implied that NlpC/P60 proteins from bacteriophages may help them penetrate the bacterial cell wall [27]. We propose that gp83 in RDJLΦ1 may have the same function based on sequence homology. ORF 84 is highly homologous to RcGTA gene 15, the largest gene encoding a single 138 kDa protein [26], which contains a rhamnosyl transferase homology and was suggested to mediate interaction between RcGTA particles and the capsule of recipient cells [28]. A 147.6 kDa homologous protein was also detected in our previous phage proteome analysis (Table 1). We inferred that this protein serves as a component of tail fibre, which is known to be involved in host specificity in broad types of tailed phages. Typical RcGTA-like gene cluster such as that in *R. capsulatus *is 15 kb long and encodes 15 gene products (Figure 2) [12,28]. Protein sequences of gp81 to gp84 in RDJLΦ1 are 35-49% identical to the corresponding sequences in RcGTA, which is the highest level of identity that we have found between RcGTA and phage sequences. Similar identity range (30-50%) was observed between RcGTA and RcGTA-like elements in other alphaproteobacteria [29]. These four RcGTA-like genes have been previously found in phage ΦJL001 (ORFs 83-86) [12]. It is noteworthy that ΦJL001 contains unmatched sequences inside ORF84 and 86 when aligned with their homologues (Figure 2, indicated by white boxes internal to blue arrows). This suggests that ΦJL001 could be relatively distantly related to RcGTA-like elements in alphaproteobacteria.

**Figure 3:**
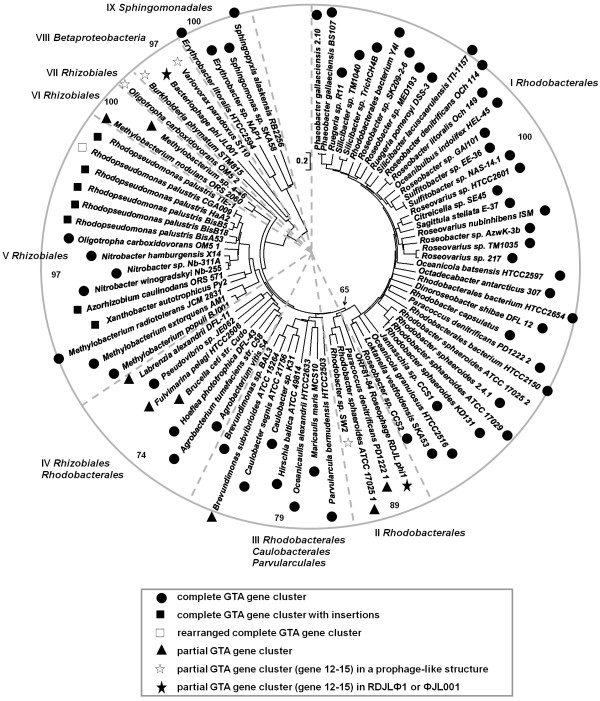
**Phylogenetic relationships of concatenated protein sequences of RcGTA-like genes 12-15 from bacteriophages and bacteria**. Amino acid sequences were aligned using Clustal X2 [31] and phylogenetic analysis was performed using the Mega 4.0 software [32]. Neighbor-joining tree was constructed using the minimum evolution distance with default parameters. Bootstrap resamplings were performed for 1,000 replications and the values (> 50%) for major branches were shown on the edge of the circular tree, except one in the center indicated by an arrow. Highly supported major subclusters were separated by dashed lines. The numbers in the end of names of bacteria (*P. denitrificans *PD1222, *R. sphaeroides *ATCC 17025 and *O. carboxidovorans *OM5) indicate multiple RcGTA-like elements in one bacterial genome. The four gene clusters in group II only contain RcGTA-like genes 12 to 15. Both PD1222 and ATCC 17025 also contain a complete RcGTA-like gene cluster (in group I). The scale bar represents 0.2 amino acid substitutions per site.

We retrieved 80 RcGTA-like gene clusters (some are partial) from 77 bacterial genomes and carried out phylogenetic analysis based on the concatenated translated sequences of RcGTA-like genes 12 to 15. The neighbor-joining tree shows that RDJLΦ1 are most closely related to *Paracoccus denitrificans *PD1222, *Rhodobacter sphaeroides *ATCC 17025 and *Rhodobacter *sp. SW2 (group II in Figure 3). We also examined the neighboring sequences of all these RcGTA-like structures. Interestingly, typical phage-like genes such as those encoding integrase, small and large terminase subunits, portal, major capsid, and tail tape measure proteins (TMP, coded by *tmp*) were identified to the left of the RcGTA-like genes from the bacterial genomes of *Rhodobacter *sp. SW2, *Burkholderia phymatum *STM815 (plasmid pBPHY01), *Variovorax paradoxus *S110 and *Oligotropha carboxidovorans *OM5 (*O. carboxidovorans *OM5 has two RCGTA-like structures, it refers to "OM5 2" here) (Figure 3). There are three evidences supporting the idea that these structures are not potential RcGTA-like gene clusters but functional elements of prophage genomes. First, no homology to RcGTA-like genes 1 to 11 was found in these gene structures. Second, other phage-like genes were found in the left arms, especially the integrase genes. Third, the *tmp *genes are much larger than those in RcGTA-like structures (2424~2739 bp vs. 660 bp). It was demonstrated that phage tail length has significant correspondence with its gene size of *tmp *[30]. These *tmp *genes in the aforementioned four bacterial genomes appear to code for components of long phage tails rather than tails of RcGTA-like particles. Lang and Beatty [12,29] suggested that RcGTA-like element is likely a remnant of a prophage ancestor that evolved to be a RcGTA progenitor in an alphaproteobacterium by losing replication, regulatory genes, and that this RcGTA progenitor then have processed a predominantly vertical descent. Partial RcGTA-like gene clusters in genuine phages and putative prophages provide evidence supporting this suggestion. It is interesting that STM815 and S110 are both betaproteobacteria. It is likely that RDJLΦ1, ΦJL001 and potential prophages in SW2, STM815, S110 and OM5 have been evolving from the proposed "prophage ancestor" and keeping the four conserved RcGTA-like genes, rather than acquired them from RcGTA-like gene clusters in alphaproteobacteria at a recent evolutionary time. They could be the "remnant" of the intermediate form on the way from "prophage ancestor" to RcGTA progenitor. Intriguingly, even a partial RcGTA-like structure and a complete one from the same bacterial genome (*e.g*. PD1222, ATCC 17025 and OM5) could diverge distantly and evolve independently (Figure 3).

The RcGTA-like gene cluster appears to consist of two modular components: (i) head-to-tail module (gene 1 to 11); (ii) tail fibre and host recognition module (gene 12 to 15). It is interesting that up to date RcGTA-like genes in a (pro)phage have been found either from region (i) or region (ii), but not from both. Likely, the juncture between regions (i) and (ii) is a hotspot for genetic recombination. The last four RcGTA-like genes seem to be a conserved element as a unit. The vertical descent inside the RcGTA-like gene clusters appears to be unbalanced that losing or keeping some element could be under certain selective pressure. However, the reason why some (pro)phages tend to retain gene module (ii) that is associated with the putative host recognition function is not clear.

## Competing interests

The authors declare that they have no competing interests.

## Authors' contributions

YZ isolated the phage, extracted the viral DNA and sequenced the genome. SH annotated the genome and carried out the phylogenetic and comparative genomic analyses. SH drafted the manuscript, and YZ, FC, NJ edited it. NJ and FC organized the study. All authors read and approved the final manuscript.

## Supplementary Material

Additional file 1 **Genes predicted from Roseophage RDJLΦ1 genome**. Click here for file

Additional file 2 **Phylogenetic analysis based on the terminase large subunit (TerL) proteins from bacteria and bacteriophages**. Click here for file
