# Supplementary material for: Complete genome sequence of a marine roseophage provides evidence into the evolution of gene transfer agents in alphaproteobacteria
Source: Virol J. 2011 Mar 17;8:124. doi: 10.1186/1743-422X-8-124 (PMC3070671; doi:10.1186/1743-422X-8-124)
Supplement: Additional file 1 — Genes predicted from Roseophage RDJLΦ1 genome. [file 1743-422X-8-124-S1.DOC]

Additional file 1 - Predicted genes of Roseophage RDJLФ1.

| ORF no. | Positions | Protein size | MW/KDa | Significant hit (Organism) | *E*-value | Putative function | Conserved domain |
| --- | --- | --- | --- | --- | --- | --- | --- |
| 1↑ | 1-432 | 144 | 15.6 | gp02 (Phage phiJL001) | 2.00E-18 |  |  |
| 2↑ | 413-688 | 92 | 10.7 |  |  |  |  |
| 3↑ | 693-1013 | 107 | 11.7 | gp04 (Phage phiJL001) | 1.00E-10 |  | PHA00649 |
| 4↑ | 1058-1987 | 310 | 34.9 | hypothetical protein (Phage PY100) | 6.00E-17 |  |  |
| 5↑ | 1977-2330 | 118 | 13.5 | hypothetical protein (*Salmonella* phage SETP3) | 1.00E-11 |  |  |
| 6↑ | 2625-2888 | 88 | 1.0 |  |  |  |  |
| 7↓ | 3009-4769 | 587 | 65.1 | ribonucleotide reductase (Phage phiJL001) | 0 | RNR, class I like family | cd02888 |
| 8↓ | 4853-5140 | 96 | 11.2 |  |  |  |  |
| 9↓ | 5137-5310 | 58 | 0.7 |  |  |  |  |
| 10↓ | 5298-5534 | 79 | 0.9 |  |  |  |  |
| 11↓ | 5544-6194 | 217 | 24.2 |  |  |  |  |
| 12↑ | 6275-6610 | 112 | 12.8 | hypothetical protein (Roseobacter sp. MED193) | 1.00E-13 |  |  |
| 13↑ | 6610-6885 | 92 | 10.7 |  |  |  |  |
| 14↓ | 6886-7350 | 155 | 17.6 | hypothetical protein (*Pseudomonas* phage LMA2) | 2.00E-06 |  |  |
| 15↓ | 7325-7840 | 172 | 19.1 |  | 3.00E-05 | Dihydrofolate reductase | COG0262 |
| 16↓ | 7831-8295 | 155 | 17.2 | deoxycytidylate deaminase  (*Xanthomonas campestris* str. ATCC 33913) | 5.00E-24 | Deoxycytidylate deaminase | COG2131 |
| 17↓ | 8292-9179 | 296 | 33.3 | thymidylate synthase (*Psychroflexus torquis* ATCC 700755) | 3.00E-20 | Thymidylate synthase | COG0207 |
| 18↓ | 9169-10215 | 349 | 39.2 | Cof (*Bacillus cereus* Rock3-44) | 1.00E-15 | Cof hydrolase | pfam01503 |
| 19↓ | 10314-11075 | 254 | 28.4 |  |  |  |  |
| 20↓ | 11068-11268 | 67 | 0.7 |  |  |  |  |
| 21↓ | 11362-11664 | 101 | 10.6 |  |  |  |  |
| 22↓ | 11763-13301 | 513 | 58.2 | Helicase (Phage phiJL001) | 3.00E-31 | Helicases | COG0553 |
| 23↓ | 13305-13664 | 120 | 14.5 | HD superfamily hydrolase (*Bdellovibrio bacteriovorus* HD100) | 2.00E-07 | Hydrolase of HD superfamily | COG1896 |
| 24↓ | 13654-13899 | 82 | 0.9 | metal dependent phosphohydrolase  (*Burkholderia pseudomallei* 1710a) | 4.00E-11 | Phosphohydrolase |  |
| 25↓ | 13911-14294 | 128 | 14.4 |  |  |  |  |
| 26↓ | 14364-14990 | 209 | 24.0 | hypothetical protein (*Solibacter usitatus* Ellin6076) | 2.00E-42 |  |  |
| 27↑ | 14993-15313 | 107 | 11.8 |  |  |  |  |
| 28↓ | 15350-17866 | 839 | 95.9 | DNA polymerase (*Yersinia pseudotuberculosis* YPIII) | 3.00E-58 | DNA polymerase A domain | smart00482 |
| 29↓ | 17870-18106 | 79 | 0.9 |  |  |  |  |
| 30↓ | 18123-18374 | 84 | 1.0 |  | 0.007 | Uncharacterized protein conserved in bacteria | COG3750 |
| 31↓ | 18371-18676 | 102 | 10.9 | hypothetical protein (*Roseovarius* sp. TM1035) | 2.00E-20 |  |  |
| 32↓ | 18802-19833 | 344 | 38.3 | nrdC.10 hypothetical protein (*Enterobacteria* phage RB14) | 7.00E-39 |  |  |
| 33↑ | 20003-20305 | 101 | 11.2 |  |  |  |  |
| 34↓ | 20391-21032 | 214 | 24.5 | hypothetical protein (*Clostridium cellulolyticum* H10) | 6.00E-14 |  |  |
| 35↓ | 21173-21700 | 176 | 19.3 | putative exonuclease (*Escherichia coli* str. 11128) | 1.00E-15 | Exonucleases | cd06127 |
| 36↓ | 21700-21921 | 74 | 0.8 |  |  |  |  |
| 37↓ | 21915-22370 | 152 | 16.6 | transcriptional regulator CtrA  (*Oceanibulbus indolifex* HEL-45) | 1.00E-18 | Transcriptional regulator | pfam00486 |
| 38↓ | 22762-23694 | 311 | 35.7 | hypothetical protein (*Roseobacter* sp. MED193) | 3.00E-16 |  |  |
| 39↓ | 23706-24041 | 112 | 12.6 | conserved hypothetical protein (*Thioalkalivibrio* sp. K90mix) | 1.00E-05 |  |  |
| 40↓ | 24034-24288 | 85 | 1.0 |  |  |  |  |
| 41↓ | 24288-24569 | 94 | 10.5 |  |  |  |  |
| 42↓ | 24566-26008 | 481 | 53.9 | conserved hypothetical protein (Bacteriophage APSE-2) | 5.00E-20 |  |  |
| 43↓ | 26009-26269 | 87 | 1.0 | hypothetical protein (*Parabacteroides distasonis* ATCC 8503) | 6.00E-06 |  |  |
| 44↓ | 26269-26478 | 70 | 0.8 |  |  |  |  |
| 45↓ | 26471-26686 | 72 | 0.8 |  |  |  |  |
| 46↓ | 27121-27597 | 159 | 16.6 | Protein TolA (*Dickeya dadantii* Ech586) | 2.00E-10 | Membrane spanning protein TolA |  |
| 47↑ | 27764-30172 | 803 | 91.3 | Integrase (*Vibrio* phage VP2) | 1.00E-50 | Integrase | cd04859 |
| 48↑ | 30234-30452 | 73 | 0.8 |  |  |  |  |
| 49↑ | 30592-30843 | 84 | 1.0 |  |  |  |  |
| 50↑ | 30840-31205 | 122 | 13.6 | hypothetical protein (*Mesorhizobium* sp. BNC1) | 5.00E-24 |  | PHA00684 |
| 51↑ | 31198-31581 | 128 | 14.2 |  |  |  |  |
| 52↑ | 31699-32037 | 113 | 12.7 |  |  |  |  |
| 53↑ | 32809-33309 | 167 | 19.0 |  |  |  |  |
| 54↑ | 33491-33895 | 135 | 15.0 |  |  |  |  |
| 55↑ | 34046-34588 | 181 | 20.5 | hypothetical protein (*Pseudomonas* phage PAJU2) | 3.00E-37 |  | COG4333 |
| 56↑ | 34895-35275 | 127 | 14.9 | endonuclease V (*Brucella suis* 1330) | 2.00E-36 | Pyrimidine dimer DNA glycosylase | pfam03013 |
| 57↑ | 35586-35855 | 90 | 10.3 | hypothetical membrane protein (*Lactobacillus delbrueckii*) | 3.00E-04 |  |  |
| 58↑ | 35840-36022 | 61 | 0.7 |  |  |  |  |
| 59↑ | 36080-36388 | 103 | 11.7 |  |  |  |  |
| 60↑ | 36407-36904 | 166 | 18.3 |  |  |  |  |
| 61↑ | 36921-37580 | 220 | 25.0 | hypothetical protein (*Rhodobacter sphaeroides* ATCC 17029) | 2.00E-62 | Putative peptidoglycan binding domain | pfam01471 |
| 62↑ | 37752-38327 | 192 | 21.8 | gp58 (Phage phiJL001) | 7.00E-05 |  |  |
| 63↑ | 38331-39689 | 453 | 51.6 | terminase large subunit (*Enterobacteria* phage ES18) | 6.00E-55 | Phage terminase large subunit | pfam04466 |
| 64↓ | 39979-40596 | 206 | 23.3 |  |  |  |  |
| 65↓ | 40659-40952 | 98 | 11.3 |  |  |  |  |
| 66↓ | 40981-41193 | 71 | 0.8 |  |  |  |  |
| 67↑ | 41363-42802 | 480 | 52.7 | hypothetical protein (*Enterobacteria* phage WV8) | 1.00E-20 |  |  |
| 68↑ | 42802-43398 | 199 | 20.7 | putative uncharacterized protein  (*Pseudomonas aeruginosa* 2192) | 3.00E-04 |  |  |
| 69↑ | 43411-43785 | 125 | 13.1 |  |  |  |  |
| 70↑ | 43786-44760 | 325 | 36.9 | putative head morphogenesis protein  (*Methylobacterium nodulans* ORS 2060) | 3.00E-36 | Head morphogenesis protein, phage Mu F protein | pfam04233 |
| 71↑ | 44852-45250 | 133 | 14.6 | hypothetical protein  (*Magnetospirillum magnetotacticum* MS-1) | 5.00E-13 |  |  |
| 72↑ | 45252-46364 | 371 | 39.4 | Mucin-associated surface protein (*Roseobacter* sp. GAI101) | 6.00E-08 |  |  |
| 73↑ | 46442-46867 | 142 | 14.2 | putative uncharacterized protein (*Roseovarius* sp. 217) | 0.001 |  |  |
| 74↑ | 46906-47940 | 345 | 37.7 | capsid protein (*Pseudomonas* phage B3) | 1.00E-07 | Major capsid protein |  |
| 75↑ | 48014-48679 | 222 | 25.3 | gp57 (*Burkholderia* phage Bcep22) | 2.00E-05 |  |  |
| 76↑ | 48682-49092 | 137 | 14.4 | hypothetical protein (*Pseudomonas* phage 73) | 4.00E-11 |  |  |
| 77↑ | 49089-49505 | 139 | 15.8 | gp77 (Phage phiJL001) | 2.00E-13 | Structural protein |  |
| 78↑ | 49523-51043 | 507 | 53.0 | major structural phage protein (*Pseudomonas* phage YuA) | 8.00E-50 | Major tail protein |  |
| 79↑ | 51129-51575 | 149 | 16.6 | hypothetical protein (*Pseudomonas* phage M6) | 7.00E-04 |  |  |
| 80↑ | 51941-55030 | 1030 | 107.9 | phage tail tape measure protein, TP901  (*Bacillus cereus* ATCC 10876) | 1.00E-50 | Tail tape measure protein | TIGR01760 |
| 81↑ | 55031-55651 | 207 | 23.2 | hypothetical protein (*Roseovarius* sp. HTCC2601) | 7.00E-46 | Putative glycoside hydrolase | TIGR02217 |
| 82↑ | 55648-56553 | 302 | 32.3 | conserved hypothetical protein  (*Hyphomicrobium denitrificans* ATCC 51888) | 2.00E-74 |  | TIGR02218 |
| 83↑ | 56522-56971 | 150 | 16.8 | phage cell wall peptidase, NlpC/P60 family  (*Caulobacter segnis* ATCC 21756) | 1.00E-34 | Phage cell wall peptidase | TIGR02219 |
| 84↑ | 56975-61060 | 1362 | 147.6 | gene transfer agent (GTA) like protein (*Rhodobacter* sp. SW2) | 0 | Putative phage tail fibre |  |
| 85↑ | 61070-62152 | 361 | 37.6 | ribonuclease III (*Brucella melitensis* 16M) | 5.00E-32 | Ribonuclease III |  |
| 86↑ | 62161-62400 | 80 | 0.9 |  |  |  |  |
| 87↑ | 62448-62648 | 67 | 0.7 | gp89 (Phage phiJL001) | 1.00E-04 |  |  |

Note: ↑ indicates + strand, ↓ indicates – strand; ORF: open reading frame; MW: molecular weight.
