# Supplementary material for: Complete genome sequence of a marine roseophage provides evidence into the evolution of gene transfer agents in alphaproteobacteria
Source: Virol J. 2011 Mar 17;8:124. doi: 10.1186/1743-422X-8-124 (PMC3070671; doi:10.1186/1743-422X-8-124)
Supplement: Additional file 2 — Phylogenetic analysis based on the terminase large subunit (TerL) proteins from bacteria and bacteriophages. [file 1743-422X-8-124-S2.PPT]

## Slide 1
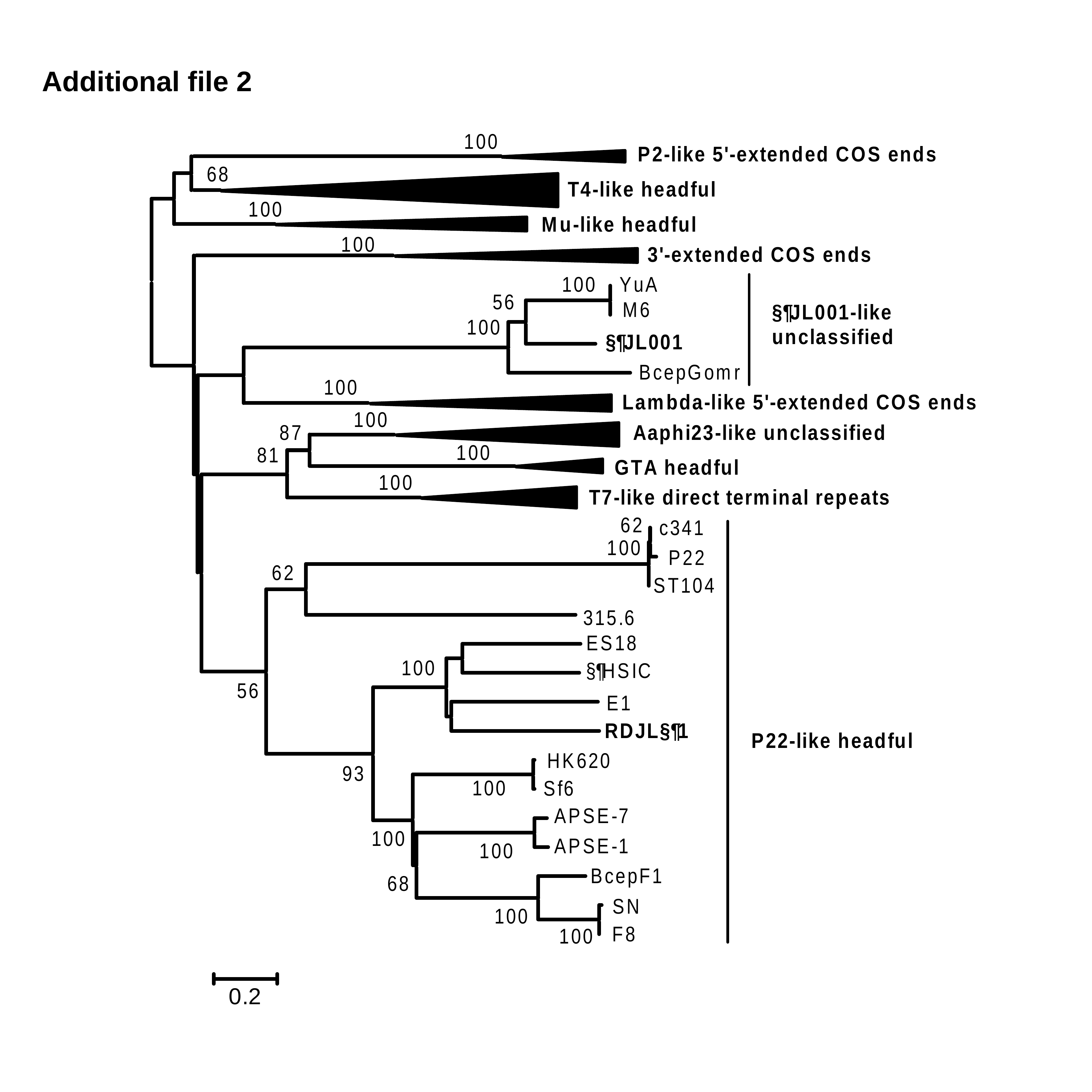

Additional file 2

## Slide 2
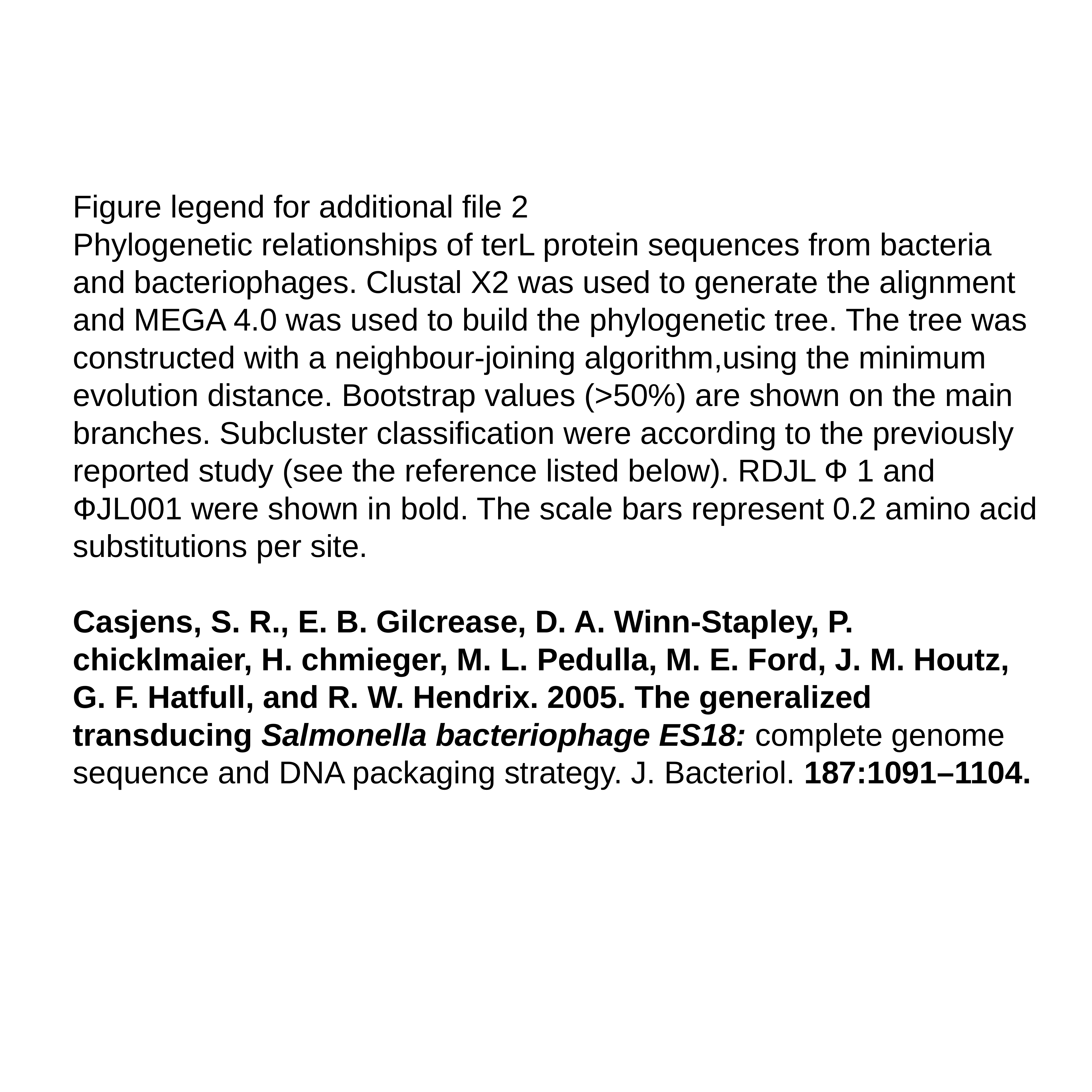

Figure legend for additional file 2
Phylogenetic relationships of terL protein sequences from bacteria and bacteriophages. Clustal X2 was used to generate the alignment and MEGA 4.0 was used to build the phylogenetic tree. The tree was constructed with a neighbour-joining algorithm,using the minimum evolution distance. Bootstrap values (>50%) are shown on the main branches. Subcluster classification were according to the previously reported study (see the reference listed below). RDJL Ф 1 and ФJL001 were shown in bold. The scale bars represent 0.2 amino acid substitutions per site.
Casjens, S. R., E. B. Gilcrease, D. A. Winn-Stapley, P. chicklmaier, H. chmieger, M. L. Pedulla, M. E. Ford, J. M. Houtz, G. F. Hatfull, and R. W. Hendrix. 2005. The generalized transducing Salmonella bacteriophage ES18: complete genome sequence and DNA packaging strategy. J. Bacteriol. 187:1091–1104.
